# Early Life Child Micronutrient Status, Maternal Reasoning, and a Nurturing Household Environment have Persistent Influences on Child Cognitive Development at Age 5 years: Results from MAL-ED

**DOI:** 10.1093/jn/nxz055

**Published:** 2019-06-04

**Authors:** Benjamin J J McCormick, Stephanie A Richard, Laura E Caulfield, Laura L Pendergast, Jessica C Seidman, Beena Koshy, Reeba Roshan, Rita Shrestha, Erling Svensen, Ladislaus Blacy, Zeba Rasmussen, Angelina Maphula, Rebecca Scharf, Baitun Nahar, Sayma Haque, Muneera Rasheed, Reinaldo Oria, Elizabeth T Rogawski, Laura E Murray-Kolb, The MAL-ED Network Investigators, Angel Mendez Acosta, Rosa Rios de Burga, Cesar Banda Chavez, Julian Torres Flores, Maribel Paredes Olotegui, Silvia Rengifo Pinedo, Mery Siguas Salas, Dixner Rengifo Trigoso, Angel Orbe Vasquez, Imran Ahmed, Didar Alam, Asad Ali, Zulfiqar A Bhutta, Shahida Qureshi, Muneera Rasheed, Sajid Soofi, Ali Turab, Anita K M Zaidi, Ladaporn Bodhidatta, Carl J Mason, Sudhir Babji, Anuradha Bose, Ajila T George, Dinesh Hariraju, M Steffi Jennifer, Sushil John, Shiny Kaki, Gagandeep Kang, Priyadarshani Karunakaran, Beena Koshy, Robin P Lazarus, Jayaprakash Muliyil, Mohan Venkata Raghava, Sophy Raju, Anup Ramachandran, Rakhi Ramadas, Karthikeyan Ramanujam, Anuradha Bose, Reeba Roshan, Srujan L Sharma, Shanmuga Sundaram E, Rahul J Thomas, William K Pan, Ramya Ambikapathi, J Daniel Carreon, Vivek Charu, Viyada Doan, Jhanelle Graham, Christel Hoest, Stacey Knobler, Dennis R Lang, Benjamin J J McCormick, Monica McGrath, Mark A Miller, Archana Mohale, Gaurvika Nayyar, Stephanie Psaki, Zeba Rasmussen, Stephanie A Richard, Jessica C Seidman, Vivian Wang, Rebecca Blank, Michael Gottlieb, Karen H Tountas, Caroline Amour, Eliwaza Bayyo, Estomih R Mduma, Regisiana Mvungi, Rosemary Nshama, John Pascal, Buliga Mujaga Swema, Ladislaus Yarrot, Tahmeed Ahmed, A M Shamsir Ahmed, Rashidul Haque, Iqbal Hossain, Munirul Islam, Mustafa Mahfuz, Dinesh Mondal, Fahmida Tofail, Ram Krishna Chandyo, Prakash Sunder Shrestha, Rita Shrestha, Manjeswori Ulak, Aubrey Bauck, Robert E Black, Laura E Caulfield, William Checkley, Margaret N Kosek, Gwenyth Lee, Kerry Schulze, Pablo Peñataro Yori, Laura E Murray-Kolb, A Catharine Ross, Barbara Schaefer, Suzanne Simons, Samuel P Scott, Laura Pendergast, Cláudia B Abreu, Hilda Costa, Alessandra Di Moura, José Quirino Filho, Alexandre Havt, Álvaro M Leite, Aldo A M Lima, Noélia L Lima, Ila F Lima, Bruna L L Maciel, Pedro H Q S Medeiros, Milena Moraes, Francisco S Mota, Reinaldo B Oriá, Josiane Quetz, Alberto M Soares, Rosa M S Mota, Crystal L Patil, Pascal Bessong, Cloupas Mahopo, Angelina Maphula, Emanuel Nyathi, Amidou Samie, Leah Barrett, Rebecca Dillingham, Jean Gratz, Richard L Guerrant, Eric Houpt, William A Petri, James Platts-Mills, Rebecca Scharf, Binob Shrestha, Sanjaya Kumar Shrestha, Tor Strand, Erling Svensen

**Affiliations:** 1 Fogarty International Center/National Institutes of Health, Bethesda, MD; 2 Johns Hopkins University, Baltimore, MD; 3 Temple University, Philadelphia, PA; 4 Christian Medical College, Vellore, India; 5 Tribhuvan University, Kathmandu, Nepal; 6 University of Bergen, Norway; 7 Haydom Lutheran Hospital, Haydom, Tanzania; 8 University of Venda, Thohoyandou, South Africa; 9 University of Virginia, Charlottesville, VA; 10 icddrb, Dhaka, Bangladesh; 11 Aga Khan University, Karachi, Pakistan; 12 Federal University of Ceara, Fortaleza, Brazil; 13 The Pennsylvania State University, University Park, PA; 14 A.B. PRISMA, Iquitos, Peru; 15 Armed Forces Research Institute of Medical Sciences, Bangkok, Thailand; 16 Duke University, Durham, NC, Fogarty International Center/National Institutes of Health, Bethesda, MD; 17 Fogarty International Center/National Institutes of Health, Bethesda, MD, Foundation for the NIH, Bethesda, MD; 18 Foundation for the NIH, Bethesda, MD; 19 Institute of Medicine, Tribhuvan University, Kathmandu, Nepal; 20 Johns Hopkins University, Baltimore, MD, Fogarty International Center/National Institutes of Health, Bethesda, MD; 21 The Pennsylvania State University, University Park, PA, Fogarty International Center/National Institutes of Health, Bethesda, MD; 22 Universidade Federal do Ceara, Fortaleza, Brazil; 23 Universidade Federal do Ceara, Fortaleza, Brazil, Fogarty International Center/National Institutes of Health, Bethesda, MD; 24 University of Illinois, Chicago, IL; 25 Walter Reed/AFRIMS Research Unit, Kathmandu, Nepal; 26 Walter Reed/AFRIMS Research Unit, Kathmandu, Nepal, University of Bergen, Norway; 27 Haukeland University Hospital, Bergen, Norway, Haydom Lutheran Hospital, Haydom, Tanzania

**Keywords:** micronutrients, diarrhea, dietary intake, illness, home environment, cognitive development

## Abstract

**Background:**

Child cognitive development is influenced by early-life insults and protective factors. To what extent these factors have a long-term legacy on child development and hence fulfillment of cognitive potential is unknown.

**Objective:**

The aim of this study was to examine the relation between early-life factors (birth to 2 y) and cognitive development at 5 y.

**Methods:**

Observational follow-up visits were made of children at 5 y, previously enrolled in the community-based MAL-ED longitudinal cohort. The burden of enteropathogens, prevalence of illness, complementary diet intake, micronutrient status, and household and maternal factors from birth to 2 y were extensively measured and their relation with the Wechsler Preschool Primary Scales of Intelligence at 5 y was examined through use of linear regression.

**Results:**

Cognitive T-scores from 813 of 1198 (68%) children were examined and 5 variables had significant associations in multivariable models: mean child plasma transferrin receptor concentration (β: −1.81, 95% CI: −2.75, −0.86), number of years of maternal education (β: 0.27, 95% CI: 0.08, 0.45), maternal cognitive reasoning score (β: 0.09, 95% CI: 0.03, 0.15), household assets score (β: 0.64, 95% CI: 0.24, 1.04), and HOME child cleanliness factor (β: 0.60, 95% CI: 0.05, 1.15). In multivariable models, the mean rate of enteropathogen detections, burden of illness, and complementary food intakes between birth and 2 y were not significantly related to 5-y cognition.

**Conclusions:**

A nurturing home context in terms of a healthy/clean environment and household wealth, provision of adequate micronutrients, maternal education, and cognitive reasoning have a strong and persistent influence on child cognitive development. Efforts addressing aspects of poverty around micronutrient status, nurturing caregiving, and enabling home environments are likely to have lasting positive impacts on child cognitive development.

## Introduction

Early childhood is a critical period for brain development and it is estimated that 250 million children worldwide are not developing optimally, with low- and middle-income countries bearing most of this burden (1). Accumulated evidence suggests that infectious diseases, nutrient deficiencies, and the home environment affect child cognitive development (2). However, the design of effective interventions for the improvement of cognitive development (3) can benefit from an understanding of which factors are persistently related to child development over time.

The focus on the first 1000 d of life has advanced our understanding of how prenatal and early postnatal environments are related to child development (4, 5). However, few previous studies have examined the persistence of early life health and nutrition insults beyond this period, including multiple simultaneous factors, pertaining to poverty, household functioning, diet, illness, and micronutrient status. More common are studies of the positive effects of caregiver and household environmental factors that provide a context for cognitive development (6, 7), or report the negative effects of summary measures of growth faltering and diarrhea rather than detailed measures of dietary intakes and infection (8–10). Indeed such studies suggest that the caregiver and household environment outweigh other factors (11). Our previous work, focused on child cognitive development from birth to age 2 y (12), revealed that symptoms of illness and the burden of enteropathogens negatively affected child cognitive development. Conversely, the safety and healthfulness of a child's environment, the child's mean B vitamin intakes from complementary foods, hemoglobin concentrations, and maternal cognitive reasoning were all positively related to child cognitive development. Here we evaluate whether those factors continued to be associated with cognitive development in the same children at age 5 y, and we re-examine factors that were originally hypothesized to relate to cognitive development at 2 y, to test whether some associations only manifest later.

We examine 3 hypotheses: (i) higher burdens of enteric infections and symptoms of illness during the first 2 y of life are associated with lower cognitive development at 5 y; (ii) a higher quality diet from complementary foods and better micronutrient status are associated with higher cognitive scores; and (iii) higher socioeconomic status and a better home environment are positively related to cognitive development at 5 y.

## Materials and Methods

The Etiology, Risk Factors, and Interactions of Enteric Infections and Malnutrition and the Consequences for Child Health and Development Project (MAL-ED) is a multidisciplinary prospective community-based birth cohort study at 8 sites in low- and middle-income countries (13). Data were collected between November 2009 and February 2017 in Dhaka, Bangladesh; Fortaleza, Brazil; Vellore, India; Bhaktapur, Nepal; Loreto, Peru; Naushero Feroze, Pakistan; Venda, South Africa, and Haydom, Tanzania. Each site enrolled and followed a cohort of ≥200 children until 24 mo of age; children were followed up at 5 y of age for the extension study. Eligible infants were enrolled <17 d old, born singleton with a birth weight >1500 g, without serious illnesses, to a mother aged ≥16 y, and to a family intending to stay in the community for ≥6 mo. The overall study design has been published along with methods of data collection from birth to 24 mo (14–17).

Each site obtained ethical approval from their respective institutions, and written consent was obtained from participants for the original study. Additional ethical approvals were obtained for the extension study by each site and a second signed consent was obtained from the participants where necessary.

### Cognitive development

The Wechsler Preschool Primary Scales of Intelligence, Third Edition (WPPSI) were used to assess cognitive abilities of study children at 5 y of age (18). The scales were adapted to account for cultural appropriateness, e.g., by removing culturally loaded verbal questions and substituting pictures or animations with locally relevant alternatives. Extensive validity analyses were conducted on the cultural adaptations (mirroring those used for the original scale development) and sample-specific norms were established (19). The test was administered by trained assessors, and ∼8% were recorded and reviewed for quality control purposes. Scores were not normalized against the US reference population because they are not representative of our sample. Following exploratory and confirmatory factor analysis, a single factor, describing child fluid reasoning (requiring reason to solve unfamiliar problems) was identified, and the structure was supported across all sites (19). This factor was derived from 3 subscales (block design, matrix reasoning, and picture completion) and loadings were converted to T-scores (i.e., pooling the data across all sites and standardizing to a mean of 50 and SD of 10). Raw cognitive development scores are not presented because of historical abuse (20).

### Maternal factors related to child development

Maternal cognitive reasoning was assessed when the child was aged 6–8 mo with the Raven's Progressive Matrices (21), and, from this, a score of fluid reasoning was calculated. Maternal depressive symptoms were measured when the child was 1, 6, 15, 24, and 60 mo with the Self Reporting Questionnaire (22). Psychometric analyses revealed a 16-item, 1-factor structure with items reflecting internalizing symptoms (23) valid for all time points (except the Brazilian data, where validity was not statistically supported) and an 18-item 1-factor solution valid at the 60-mo time point.

### Illness and microbiology

Methods for twice-weekly disease surveillance and monthly assessment of pathogens in stool during early childhood have been published previously (14, 15, 24). At the 5-y visit, households were asked whether the child had been ill or had required hospitalization in the previous 30 d.

Nondiarrheal stool samples were collected and analyzed monthly in the first year of life and quarterly in the second, to characterize exposure to >40 pathogens (24). Stools were recently re-analyzed through use of compartmentalized, probe-based quantitative PCR with custom TaqMan Array Cards (ThermoFisher), as described elsewhere (25).

### Gut function and permeability

Gut health influences nutrient absorption and metabolism, but can be affected by enteric infections, and, therefore, we collected markers of gut function and permeability. From the monthly nondiarrheal stools, 3 biomarkers were analyzed to characterize gut function: myeloperoxidase, neopterin and α-1-antitripsin (26). Following previous work (27), linear mixed models were constructed to model gut function biomarker concentrations as a function of age, stool consistency and duration of storage, recent (≤7 d) systemic inflammation (with maternally reported fever as proxy), and breast milk consumption. The residuals of these models (i.e., having de-trended the concentrations) were retained as adjusted values for further analysis.

The lactulose: mannitol (LM) test was administered at 3, 6, 9, and 15 mo to evaluate intestinal permeability (26). Values were normalized to a reference (Brazil) to remove age, sex, and laboratory trends, and results are expressed as *z* scores (LMZ) (28).

### Nutrition and micronutrient status

At enrolment, length and weight were assessed (29). Caregivers were asked about feeding starting at birth, and were interviewed twice weekly about breastfeeding and nonbreast milk food consumption. From 9 to 24 mo, intake of complementary foods was quantified monthly with use of the 24-h recall method. We adjusted Box-Cox transformed nutrient intakes for variation in energy intake with use of the residual method to create standardized nutrient densities (30).

To characterize micronutrient status, venous blood samples were collected at 7, 15, and 24 mo, and finger prick blood samples were obtained to determine hemoglobin concentration via HemoCue (31). Plasma retinol (high-performance liquid chromatography) and zinc (atomic absorption spectrophotometry) were determined along with plasma ferritin and transferrin receptor (TfR) (immunoturbidimetric [BGD and PKN; Hitachi 902 analyzer], chemiluminescent [INV; Hitachi 912 analyzer] or enzyme immunoassay [all other sites; Ramco Laboratories Inc.]). Plasma α-1-acid glycoprotein concentrations were measured (with autoanalyzers as above, or radial immunodiffusion; Kent Laboratories) to assess systemic inflammation and to adjust the ferritin and TfR concentrations (32). Hemoglobin was adjusted for altitude as appropriate (33). For analyses, we calculated the mean of each child's biochemical indicators from all available blood draws and used square-root transformations to normalize their distributions where appropriate.

### Socioeconomic status and the home environment

Families were asked about household assets and income, type of sanitation, source of drinking water, and hand-washing behaviors, as well as maternal education at 6-mo intervals. A socioeconomic index, the Water, Assets, Maternal education and household Income, was constructed through use of the sum of each equally weighted subscale and the mean score of each site used to adjust for socioeconomic differences (34).

The Black et al. modification (35) of the infant/toddler version of the Home Observation for Measurement of the Environment (HOME) Inventory (36) was used at 24 mo to capture dimensions of the home environment beneficial for child development. Three factors supported by psychometric analysis of the survey at age 24 mo were examined, describing the safety and healthfulness of the environment around the child (4 items including, e.g., the house was relatively light and the stove located in a relatively safe area), the child's cleanliness (4 items, e.g., that the child was relatively clean and the child's hair was relatively clean), and their emotional and verbal responsivity (with 13 items including whether the caregiver spontaneously praised the child's behavior or spontaneously vocalized to the child) (37).

Caregiver report of child school attendance was collected during the follow-up period and was dichotomized in these analyses as any (daycare, preschool, kindergarten, and/or primary school) or none (no schooling).

### Sample size

Overall, 1842 infants were enrolled from the 7 sites included in these analyses; WPPSI data were not available from the Peru site. Of these, 1518 remained in the study at 24 mo and 1198 (79%) had a WPPSI assessment at 60 ± 2 mo. Minimum numbers of observations per child were required for inclusion in the analysis: ≥700 d of surveillance for illness, ≥9 surveillance stools assessed for enteropathogens and gut function variables, ≥11 dietary 24-h recall measures, a 24-mo HOME assessment, a Raven's assessment, and blood biomarkers assessed at least once. These requirements, based on visual assessment of distributions of observed data and an effort to balance the representation of any 1 child (e.g., contributing an approximately equal number of observations for a given variable) with the number of children included in the analysis, reduced the sample size to 813. Characteristics of children included in the analyses (required to have data on all variables in the model) were compared to the larger group who were tested at 5 y and to those tested at 2 y.

### Statistical analyses

Candidate variables from the 0 to 24 mo data collection were identified from our previously constructed conceptual model of early cognitive development (12); in addition, variables collected from 24 to 60 mo were also considered, based on domains that were included in our original model. Given the multiple ways to characterize any particular domain, the long-list of candidate variables (**Supplemental Table 1**) was filtered through use of univariate linear mixed models with the WPPSI T-score as the outcome and including a random intercept for the child's site. Variables with *P *≤ 0.2 were retained.

**FIGURE 1:**
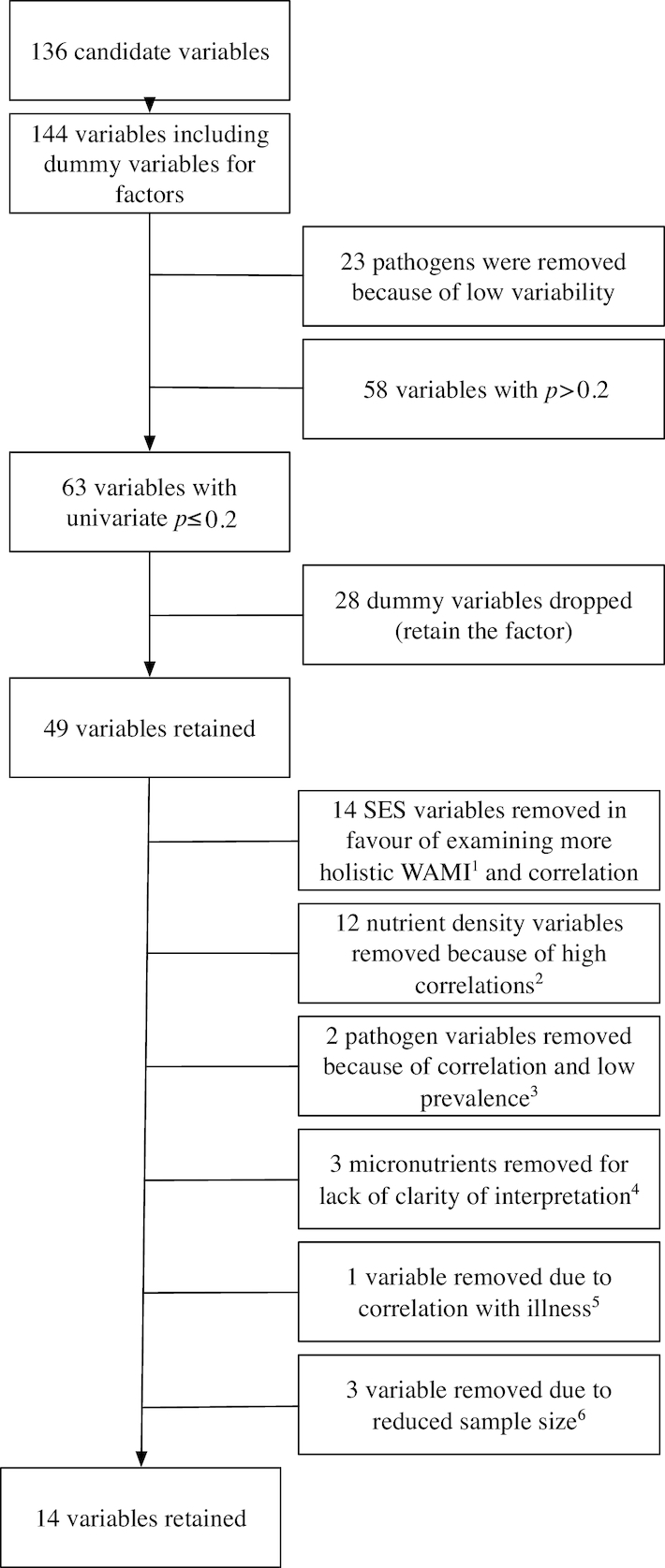
Selection of candidate variables from data collected in children and their mothers in The Etiology, Risk Factors, and Interactions of Enteric Infections and Malnutrition and the Consequences for Child Health and Development Project (MAL-ED). ^1^Ownership of agricultural land, ownership of a separate kitchen, floor and roof materials, the type of stove, water treatment and source of drinking water and hygiene, hand washing after going to the toilet, before food preparation and after the child defecates, mean monthly household income, years of maternal education, and the number of a defined list of assets. The HOME environmental safety variable was highly correlated with the child cleanliness variable and therefore dropped. ^2^Calcium, copper, iron, manganese, magnesium, potassium, and phosphorous, vitamins A, C, and E, total fat and saturated fats, protein from dairy and carbohydrate. ^3^Surveillance detections of *A. lumbricodes* (low prevalence), and the total number of pathogens because of correlation with bacterial pathogens. ^4^The number of observations of anemia, high transferrin receptor or low ferritin. ^5^The proportion of days with antibiotic given. ^6^The child's weight at 60 mo and at enrolment and the mother's depressive symptoms (from 6 to 8 mo). Anemia at 60 mo was also assessed, but was not significant in the univariate model.

Correlations (Spearman's rank, ρ) between candidate variables were examined for co-linearity. Where variables were correlated (e.g., ρ > 0.4), biological justifications and support in the literature were used to preferentially retain 1 variable (**Figure 1**).

A multivariable model was constructed to address our 3 hypotheses based on the domains identified in our conceptual model (**Supplemental Figure 1**), with the variables retained after the univariate filtering and the WPPSI T-score as the outcome, with *P *≤ 0.05. Given the interconnectedness of each domain in the conceptual model, the variables included in the multivariable model were representative of key exposures in the 3 hypotheses (enteropathogen infection, illness, diet and micronutrient status, and socioeconomic status). This conceptual model allowed identification of potential confounding between pathways. Only maternal reasoning was a confounding factor (to the HOME environment) and not explicitly one of our original hypotheses. Site was included to account for differences across the population mean WPPSI scores and similarly structured models were also constructed for each site to examine differences in patterns of coefficients. Following the prior conceptual model, interaction terms were used to test (*P < *0.05) hypothetical moderating relations between socioeconomic variables (income, assets, and maternal education) and maternal reasoning and more proximal variables in the model. To avoid overfitting, least absolute shrinkage and selection operator (LASSO) regression with partial-ridge bootstrapping was used to generate 95% CI (38). To examine the robustness of the model to missing observations, multiple imputation chain equations were used including all children with a valid WPPSI assessment (39).

## Results

The number of children with complete data for covariates differed by site, in particular for the HOME evaluations, Raven's progressive matrices, and blood biomarkers (**Supplemental Figure 2**). Compared to the children in the analytic sample, those excluded had higher mean intakes of energy and multiple micronutrients from complementary foods from age 9 to 24 mo. Their mothers also reported the child as being ill less frequently, but differences were small.

All of these children were growing up in a context of poverty but most of the mothers had at least an elementary school education, with the exception of those in Pakistan (**Table 1**). Many of the children experienced illness for a substantial proportion of days during their first 2 y and most had at least 1 pathogen detected in nondiarrheal stools each month, the majority of which were bacterial. Micronutrient intakes from complementary foods varied by site but, on average, were low.

**TABLE 1:** Environmental factors (included in the multivariable model) measured during the first 2 y of life by site in the MAL-ED project (*n *= 813)^1^

Variable	Dhaka, Bangladesh (*n *= 160)	Vellore, India (*n *= 173)	Bhaktapur, Nepal (*n *= 113)	Naushero Feroze, Pakistan (*n *= 153)	Fortaleza, Brazil (*n *= 49)	Venda, South Africa (*n *= 109)	Haydom, Tanzania (*n *= 56)
Maternal factors							
Maternal reasoning (Raven's score, 6–8 mo)	13 (10, 22)	34 (28, 40)	34 (28, 40)	14 (8, 33)	36 (32, 41)	35 (29, 40)	38 (36, 42)
Child health							
Maternally reported illness (proportion of d, 0–24 mo)	0.52 (0.42, 0.64)	0.41 (0.28, 0.53)	0.14 (0.10, 0.21)	0.78 (0.64, 0.88)	0.042 (0.03, 0.07)	0.04 (0.02, 0.07)	0.09 (0.06, 0.13)
Pathogen detection rate (detections/surveillance stool)	1.10 (0.94, 1.30)	1.00 (0.86, 1.40)	0.76 (0.62, 1.00)	1.60 (1.30, 1.90)	1.20 (0.93, 1.40)	0.69 (0.50, 0.86)	1.30 (1.10, 1.60)
Bacterial pathogen detection rate (detections/surveillance stool)	0.92 (0.75, 1.10)	0.79 (0.62, 1.00)	0.62 (0.50, 0.79)	1.10 (0.93, 1.30)	1.00 (0.86, 1.20)	0.50 (0.38, 0.64)	1.00 (0.81, 1.20)
NEO (nmol/L)^2^	6.9 (6.6, 7.2)	7.4 (7.2, 7.7)	7.3 (7.2, 7.5)	6.2 (6.0, 6.4)	7.4 (7.2, 7.6)	8.2 (8.0, 8.4)	6.6 (6.3, 6.9)
TfR (mg/L)^2^,^3^	5.8 (4.6, 8.2)	4.5 (3.5, 6.3)	8.8 (7.2, 12.0)	4.9 (3.6, 7.8)	9.5 (7.7, 11.0)	4.2 (2.7, 5.8)	4.3 (3.6, 6.1)
Diet from complementary foods							
Total energy (kcal/d)	330 (270, 400)	730 (600, 880)	420 (340, 520)	640 (490, 780)	980 (840, 1100)	880 (750, 1000)	990 (880, 1100)
Animal source protein (g/d)	3.5 (2.4, 5.1)	8.2 (5.3, 15.0)	5.0 (3.2, 7.7)	7.3 (4.4, 11.0)	33.0 (29.0, 39.0)	11.0 (8.5, 14.0)	9.7 (5.0, 14.0)
Vitamin A (μg/d)	52 (36, 77)	180 (120, 280)	85 (61, 110)	160 (100, 240)	930 (750, 1100)	370 (270, 540)	130 (93, 180)
Vitamin B-12 (μg/d)	0.36 (0.25, 0.57)	0.62 (0.41, 0.92)	0.51 (0.31, 0.82)	0.73 (0.46, 1.30)	4.60 (3.90, 5.40)	1.10 (0.60, 1.60)	1.10 (0.61, 1.70)
Vitamin D (μg/d)	1.1 (0.7, 1.5)	0.1 (0.0, 0.2)	0.1 (0.1, 0.2)	0.1 (0.1, 0.1)	5.4 (4.1, 7.1)	1.2 (0.7, 2.4)	0.0 (0.0, 0.0)
Zinc (mg/d)	1.1 (0.9, 1.4)	2.8 (2.3, 3.7)	1.4 (1.1, 1.8)	2.0 (1.5, 2.6)	9.0 (7.1, 11.0)	6.1 (4.9, 7.3)	5.2 (4.7, 5.9)
Iron (mg/d)	1.3 (1.0, 1.7)	2.3 (2.1, 2.8)	1.3 (1.1, 1.7)	2.0 (1.7, 2.7)	13.0 (11.0, 17.0)	7.9 (6.8, 9.9)	8.1 (6.9, 9.0)
Socioeconomic and home environment							
HOME child cleanliness score (24 mo)	4 (4, 4)	3 (2, 4)	4 (4, 4)	3 (1, 4)	4 (3, 4)	4 (4, 4)	3 (1, 3)
Mean monthly household income (USD)	120 (91, 150)	72 (58, 98)	130 (100, 210)	140 (91, 220)	350 (320, 420)	240 (180, 390)	21 (15, 37)
Maternal education (y)	5 (2, 7)	8 (4, 9)	10 (6, 12)	2 (0, 5)	10 (8, 12)	11 (9, 12)	7 (3, 7)

1 Values are median (25th, 75th percentiles). HOME, Home Observation for Measurement of the Environment; MAL-ED, The Etiology, Risk Factors, and Interactions of Enteric Infections and Malnutrition and the Consequences for Child Health and Development Project; NEO, neopterin; TfR, transferrin receptor.

2 Log scale.

3 Adjusted for inflammation.

### Univariate associations

Relations between WPPSI fluid reasoning T-scores and variables characterizing enteropathogens, illness, anthropometric status, diet and micronutrient status, gut function, home environment, and maternal cognitive domains were tested; across all domains, 63 variables were retained based on our predefined filtering threshold (*P < *0.2, Figure 1 and Supplemental Table 1).

The mean total number of pathogens and of bacterial pathogens detected per stool were each associated with a lower WPPSI score (β: −1.60, 95% CI: −2.84, −0.36; *P = *0.01 and β: −2.19, 95% CI: −3.88, −0.503; *P = *0.01, respectively). Of individual pathogens examined, only *Campylobacter* and *Ascaris lumbricodes* were related to the WPPSI in the univariate models although *Ascaris* detections were uncommon (285 out of 25,583 positive monthly stools), resulting in wide confidence intervals. Of the gut function markers, only mean neopterin met our criterion to be retained (β: −1.07, 95% CI: −2.62, 0.47; *P = *0.17).

Of the illness variables, fieldworker-confirmed diarrhea or acute lower respiratory infections (ALRI) were not associated with the WPPSI (β: −1.07, 95% CI: −12.56, 10.42; *P = *0.86 and β: 0.88, 95% CI: −7.44, 9.19; *P = *0.87, respectively) although both the proportion of days when the child received antibiotics (β: −6.14, 95% CI: −14.67, 2.39; *P = *0.16) and the proportion of days that the mother reported child illness (β: −2.67, 95% CI: −6.15, 0.82; *P = *0.13) met the criterion to be retained.

For many nutrients, variation in the nutrient density of the diet from nonbreast milk foods was statistically related to the WPPSI in the univariate analyses. However, strong correlations exist between various nutrient densities because of the way in which nutrients exist within foods and by meal patterns. For further analyses, we focused on micronutrients associated with animal-source proteins, indicative of greater diet quality and diversity, which consistently tended to be positively associated with the WPPSI. Usual intakes of vitamin B-6 and folate from nonbreast milk foods were associated with higher cognitive development scores at 24 mo (12), but they were not found to be associated at 5 y (β: −0.18, 95% CI: −0.84, 0.48; *P = *0.59 and β: 0.36, 95% CI: −0.39, 1.1; *P = *0.35, respectively). Breastfeeding was nearly universal in this cohort, and most children were breastfed to ≥18 mo; variation in breastfeeding practices was not found to be associated with WPPSI at age 5 y.

Mean hemoglobin concentration (across as many as 3 early blood draws) had no relation with the WPPSI (β: 0.15, 95% CI: −0.26, 0.56; *P = *0.50), nor did concurrent hemoglobin concentration (β: −0.08, 95% CI: −0.52, 0.36; *P = *0.72) or anemia observed at 5 y (β: −0.20, 95% CI: −1.86, 1.46; *P = *0.81). Child anemia (observed at any of 7, 15, or 24 mo) was negatively related to the WPPSI (β: −1.71, 95% CI: −2.81, −0.60; *P = *0.03). Mean TfR concentration was negatively associated with the WPPSI (β: −1.45, 95% CI: −2.34, −0.63; *P < *0.01).

The Water, Assets, Maternal education and household Income index was strongly positively associated with the WPPSI score (β: 15.6, 95% CI: −12.3, 18.9; *P < *0.01), and several subcomponents—household income (β: 0.007, 95% CI: 0.003, 0.01; *P < *0.01), assets (β: 1.13 95% CI: 0.86, 1.41; *P < *0.01), and maternal education (β: 0.55, 95% CI: 0.42, 0.68; *P < *0.01)—were similarly associated. Both child cleanliness and household safety measured with the HOME survey were positively associated with the WPPSI scores (i.e., the cleaner the child [β: 1.07, 95% CI: 0.60, 1.54; *P < *0.01] and safer the environment [β: 1.02, 95% CI: 0.42, 1.62; *P < *0.01], the higher the WPPSI scores). Maternal cognitive reasoning (β: 0.16, 95% CI: 0.11, 0.21; *P < *0.01) and child school attendance (β: 2.41, 95% CI: 0.96, 3.86; *P < *0.01) were also positively related to the WPPSI score, and maternal depressive symptoms (at 24 mo) were negatively associated with the WPPSI (β: −0.17, 95% CI: −0.32, −0.01; *P = *0.03).

### Multivariable associations

**FIGURE 2:**
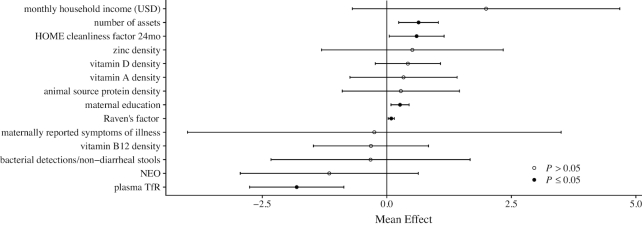
Results of the multivariable model (*n *= 813 children) showing the mean effects and 95% CI for associations between early life risk factors and cognitive development scores at age 5 y in children from The Etiology, Risk Factors, and Interactions of Enteric Infections and Malnutrition and the Consequences for Child Health and Development (MAL-ED) Project. HOME, Home Observation for Measurement of the Environment; NEO, neopterin; TfR, transferrin receptor.

**FIGURE 3:**
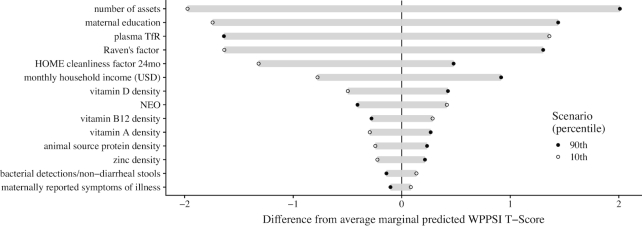
Difference in the predicted marginal difference in Wechsler Preschool and Primary Scale of Intelligence (WPPSI) scores at 5 y if individual variables are set at their respective 10th (open circle) or 90th (closed dot) observed percentile compared to the marginal mean. HOME, Home Observation for Measurement of the Environment; NEO, neopterin; TfR, transferrin receptor.

Of the variables retained after the univariate analyses, 5 were significantly related to the WPPSI T-scores in our multivariable model (**Figure 2** and **Supplemental Table 2**): assets score (β: 0.64, 95% CI: 0.24, 1.04, *P *< 0.01), HOME child cleanliness factor (β: 0.6, 95% CI: 0.05, 1.15, *P *= 0.03), years of maternal education (β: 0.27, 95% CI: 0.08, 0.45, *P *< 0.01), maternal cognitive reasoning (β: 0.09, 95% CI: 0.03, 0.15, *P *< 0.01), and mean plasma TfR concentration (β: −1.81, 95% CI: −2.75, −0.86, *P *< 0.01). There was some variability in the mean effects between sites, in particular for dietary variables that reflect substantially different intakes (**Supplemental Figure 3** and Table 1). The marginal effects of setting individual variables to their observed 10th or 90th percentiles are shown in **Figure 3**.

Contrary to our original hypotheses, after adjusting for other factors, there was no statistical support (*P = *0.75) for an association between early exposure (0–2 y) to enteric pathogens and the WPPSI score at 5 y. Bacterial pathogens were negatively but not significantly associated with the WPPSI score (β: −0.33, 95% CI: −2.33, 1.67). Total pathogen detection rate diluted the signal, i.e., the association was weaker and positive (**Supplemental Table 3**). More recent quantitative PCR data showed similar patterns for the total bacterial enteropathogen burden, albeit with a smaller mean effect (β: −0.01, 95% CI: −1.55, 1.52, *P *= 0.98) (Supplemental Table 3). After adjustment, we also found no associations between aspects of child dietary intake and WPPSI scores at 5 y, although, as stated above, we did find associations with micronutrient status.

Subanalyses were performed because the inclusion of some variables of interest would substantially reduce the number of children included (**Supplemental Table 4**). Maternal depressive symptoms, although supported at the univariate level were not related to the WPPSI in the multivariable model (β: −0.1, 95% CI: −0.27, 0.07, *P *= 0.23, *n = *734). Schooling attendance was also not supported in a multivariable model (β: 1.51, 95% CI: −0.14, 3.16, *P *= 0.07, *n = *797), largely because of homogeneity at individual sites whereby all children received (or did not) some level of schooling.

Weight-for-age at enrolment (classified into tertiles) had been an important modifier of the relations in the previous 24-mo model of cognitive development (12), and was tested for inclusion here. The only interaction term that was statistically significant (*P < *0.05) was for children in the highest weight tertile who had a larger positive effect of higher maternal cognitive reasoning than children with the reference (middle tertile) or lowest tertile enrolment weights. In general, interaction terms between household characteristics (income, maternal education, and HOME scores) and other factors were, with one exception, not statistically supported in the model and did not improve the fit of the model (with use of Akaike information criterion (AIC)). The exception was an interaction between the HOME child cleanliness factor and maternal education, indicating that the positive association of maternal education with WPPSI was weaker in environments with lower child cleanliness scores. This relation was only weakly supported, however, as the data were sparse for some combinations of both variables and were clustered within individual sites.

To correct for overfitting, we repeated the multivariable model with use of least absolute shrinkage and selection operator regression and to account for missing values, we used multiple imputation chain equations. Neither of these changed the interpretation of any results (**Supplemental Table 5**), but the negative effects of bacterial pathogens (β: −1.26, 95% CI: −3.01, 0.48, *P *= 0.20, *n = *1198) and of illness (β: −0.93, 95% CI: −4.31, 2.46, *P *= 0.58, *n = *1198) were larger in the analyses with imputed values.

## Discussion

The in-depth data collection during early childhood in the MAL-ED study with follow-up at 5 y of age allowed us to examine long-term impacts of a wide variety of early life influences on cognitive development in a manner that has not been possible in most studies to date. Our results, concordant with previous studies (6, 7), indicate that early life iron status and home environmental measures had strong and positive persistent consequences on cognitive development in later childhood. In our previous analysis, hemoglobin concentration was positively related to cognitive development scores at 2 y; the present analysis at age 5 y reveals continued effects of iron status on cognitive development as indicated by the significant negative association of TfR concentration with cognitive scores. Higher burdens of enteropathogens and illness, which were significantly negatively related to cognitive development scores at 2 y, continued to be negatively related at 5 y, but their continued effects were not statistically supported in the multivariate analysis. In addition, there was no evidence that the micronutrient densities of complementary foods continued to have a positive association with cognitive development as assessed by the WPPSI scores at 5 y. Thus, from our 3 study hypotheses, we found evidence to support a persistent, positive association between better iron status and cognitive scores as well as a better home environment and other aspects of socioeconomic status and cognitive scores at 5 y.

At age 2 y, we found that illness symptoms mediated the relation between pathogens and cognitive development, but we did not find an association between early life symptoms of illness and 5-y WPPSI scores. This is contrary to our hypothesis and also contrasts with earlier studies that showed negative effects of diarrhea on school readiness (10). Similarly, we found no statistical evidence of a persistent association between early life enteropathogen infections and cognitive development at 5 y (even accounting for the different rates of illness observed between sites). A relation was detected at 2 y, but by 5 y the relation, although negative on average, was not statistically significant. Because the hypothesized negative effect of enteropathogens on cognitive development is thought to be driven, in large part, by inflammation, we more closely examined our pathogen data, concentrating on bacterial pathogen burden, imputing missing data, and with use of more recent quantitative PCR results, but in all cases the relation with WPPSI was not statistically significant when measures of household wealth (either income or assets) were included.

Similarly, there was evidence from our earlier work that B vitamins, and other micronutrients associated with animal-source foods were positively associated with cognitive development, but the hypothesized association between early life intakes and 5-y cognitive scores were not significant in our multivariable model. There was a wide range in nutrient intakes by site and, in those sites with a higher consumption of animal-source foods, the association with our outcome was stronger. Our earlier findings fit within a literature on the advantages of animal-source foods, which synergistically provide micronutrients (40, 41). Although we did not find a persistent association of early life intakes, it may be that concurrent intake of animal-source foods is more closely related to the cognitive scores. Neither our earlier work nor our results here support a relation between breastfeeding and cognitive development, despite previous reports of such (42, 43). This should not be misinterpreted as evidence that there is no long-term benefit of breastfeeding, as nearly all of the mothers chose to breastfeed.

A novel finding, consistent across the sites, was that higher mean concentration of plasma TfR from birth to 24 mo was associated with lower WPPSI scores at 5 y. Although not focused on TfR concentrations per se, previous studies have indicated a relation between early life iron deficiency anemia and later cognitive development (44, 45). Most studies to date have used anemia as a nonspecific indicator of iron status and many report associations with cognitive development, including our earlier analysis at 24 mo (12). The lack of association between mean hemoglobin concentration and the 5-y WPPSI scores in this analysis was unexpected, but, coupled with the TfR findings, may implicate iron deficiency as the related factor rather than anemia, which can reflect multiple conditions and, in these populations, may result from other nutrient deficiencies and infections. TfR concentrations are not only affected by iron deficiency, but also by inflammation under certain circumstances (although we adjusted for this) and by cellular proliferation, which in early childhood is associated with erythropoiesis and rapid growth. In our analyses of growth outcomes at 5 y, mean TfR concentration was positively associated, whereas mean ferritin concentration was negatively associated with height-for-age *z* (HAZ) and weight-for-age *z* (WAZ), and mean hemoglobin concentration was positively related to HAZ alone (46). Given the generally low iron intakes of these children, the conflicting results for growth and development outcomes at 5 y may suggest a trade-off, as has been demonstrated in both animal and human studies under conditions in which iron supply does not meet the demand (47, 48). In these circumstances, iron is prioritized to red blood cells over the brain and, although the cellular mechanisms are unknown, it has been hypothesized to occur through increased expression of TfR on red cell precursors and decreased expression of TfR in the brain (47). Our measurements of TfR are in the periphery and, thus, may reflect increased expression on red cell precursors.

Our results indicate that cognitive development has persistent associations with socioeconomic status, but also shed light on subtleties beyond poverty. Indeed the proximal acquired insults of infection and lower quality diet that were related to early cognitive function at 24 mo, even controlling for the household context, no longer had significant associations, whereas the micronutrient status, household, and family factors continued to be important at age 5 y. Although maternal education and cognitive reasoning are related, both were independently informative. Maternal cognitive reasoning (and notably the ability to solve novel problems [fluid reasoning]) has been shown to influence child cognitive development through pathways related to household socioeconomic status and aspects of the home environment (49, 50), in addition to pathways related to the nature of mother-child interactions, including hygiene practices (51). The HOME variables are noteworthy both as observations of specific characteristics of the household environment and as characteristics contributing to a wider conceptual construct. The HOME scores that were significant in our analyses represent just 2 aspects of the home environment, but are consistent with a literature on the positive benefits of a nurturing (51), stimulating (52), and enabling environment (53) on child development (49, 54). We found some statistical evidence of an interaction between maternal education and the HOME child cleanliness score, suggesting a weaker association of education with WPPSI in settings with lower child cleanliness scores. Although significant, the combination of more maternal education and low child cleanliness score within a household was unusual in these data (for children with low HOME cleanliness scores, more than half had mothers with ≤5 y of education, and <5% had >10 y). Therefore, the interaction may be a consideration in distinguishing between education and health-promoting behaviors, but more targeted work would be needed to understand their meaning across these and other settings.

This study benefited from consistent data collection from a wide range of domains during the first 2 y of life from multiple, contrasting populations. The aim here was the identification of patterns that were consistent across these populations; however some variables were markedly different between populations, e.g., complementary diets or rates of illness, and therefore the relative importance of these factors varied by site. Primarily because of the gap between funding the original study and the extension, many children were lost to follow-up and this differentially impacted the sample size at each site. The loss of several sites because of psychometric differences or availability of data was regrettable, but ensured that those sites retained in the analyses had robust and comparable data.

In conclusion, we identified associations between early life micronutrient status, maternal education and cognitive reasoning, household wealth, and home environmental measures with cognitive development at age 5 y. Thus, improvements in cognitive function are more likely to be gained from interventions targeting the socioeconomic context of societal development and our results suggest directions for interventional efforts that address aspects of poverty around micronutrient status, the nurturing role of caregivers with or without increases in formal education, and developing safe and enabling home environments.

## Supplementary Material

nxz055_Supplemental_File

## Notes

The Etiology, Risk Factors and Interactions of Enteric Infections and Malnutrition and the Consequences for Child Health and Development Project (MAL-ED) is carried out as a collaborative project supported by the Bill & Melinda Gates Foundation, the Foundation for the NIH, and the National Institutes of Health/Fogarty International Center. This work was also supported by the Fogarty International Center, National Institutes of Health (D43-TW009359 to ETR).

Author disclosures: BJJM, SAR, LEC, LLP, JCS, BK, RR, RS, ES, LB, ZR, AM, RS, BN, SH, MR, RO, ETR, and LEM-K, no conflicts of interest.

Supplemental Tables 1–5 and Supplemental Figures 1–3 are available from the “Supplementary data” link in the online posting of the article and from the same link in the online table of contents at https://academic.oup.com/jn/.

Abbreviations used: HOME, Home Observation for Measurement of the Environment inventory; MAL-ED, The Etiology, Risk Factors, and Interactions of Enteric Infections and Malnutrition and the Consequences for Child Health and Development Project; TfR, transferrin receptor; WPPSI, Wechsler Preschool Primary Scales of Intelligence.
