# Supplementary material for: Early Life Child Micronutrient Status, Maternal Reasoning, and a Nurturing Household Environment have Persistent Influences on Child Cognitive Development at Age 5 years: Results from MAL-ED
Source: J Nutr. 2019 Jun 4;149(8):1460–9. doi: 10.1093/jn/nxz055 (PMC6686051; doi:10.1093/jn/nxz055)
Supplement: nxz055_Supplemental_File [file nxz055_supplemental_file.pdf]

Supplementary Data

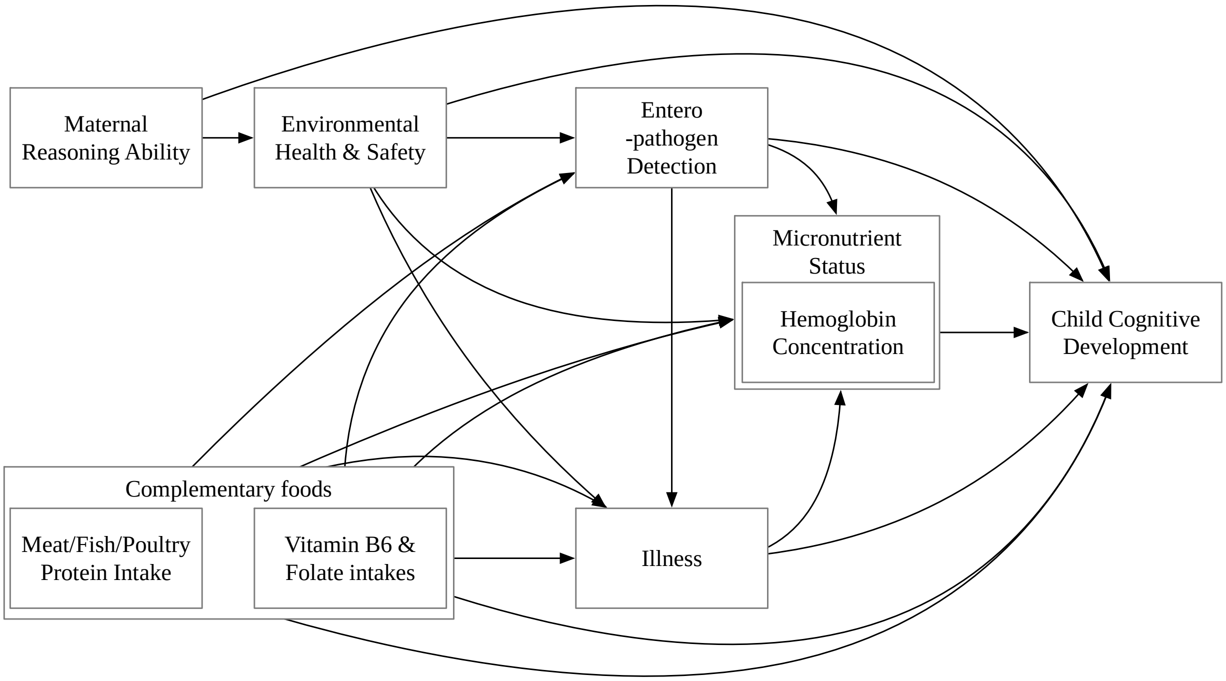

Supplemental Figure 1: Conceptual model based on (1). Confounding was identified using the rules established in (2). Given the three hypotheses examined here (namely, (i) negative effects of enteropathogens and illness, (ii) positive effects of complementary foods and micronutrient status and (iii) the positive effects of higher socio-economic status [represented by environmental health and safety]), maternal reasoning considered as a confounding factor.

	BGD	INV	NEB	PKN	BRF	SAV	TZH	Total
Followed to 24mo	209	226	228	248	165	239	203	**1518**
WPPSI	193	212	126	195	115	193	164	**1198**
with SES	193	210	126	195	115	192	164	**1195**
with ≥700d illness surveillance	193	210	126	195	115	187	164	**1190**
with ≥9 surveillance stools for pathogens	193	207	126	184	89	187	164	**1150**
with ≥9 surveillance stools for EE biomarkers	193	206	125	165	88	184	164	**1125**
with ≥11 24h recall for complementary feeding	193	206	125	165	88	170	157	**1104**
with 24mo HOME survey data	185	204	124	163	80	153	129	**1038**
with maternal Raven's assessment	181	173	123	160	53	127	89	**906**
with blood biomarkers	160	173	113	153	49	109	56	**813**

Supplemental Figure 2: Number of children followed to 24 months and then those retained for analysis based on the inclusion of different putative covariates from 0-24 months of life in the MAL-ED project; BGD: Bangladesh – Dhaka; INV: India – Vellore; NEB: Nepal – Bhaktapur; BRF: Brazil – Fortaleza; PEL: Peru – Loreto; SAV: South Africa – Venda; TZH: Tanzania – Haydom

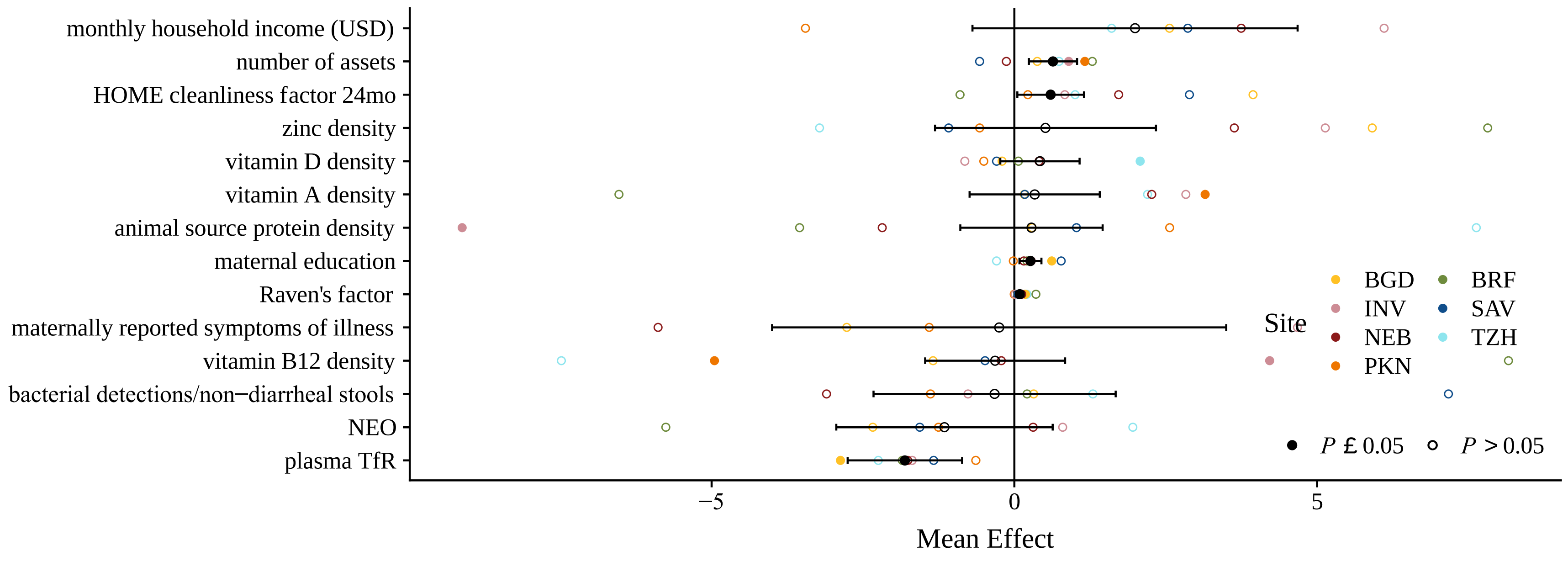

Supplemental Figure 3: Results of the multivariable model (*n*=835 children, black symbols) showing the mean effects and 95% confidence intervals. The mean effects from site-specific models are illustrated in colored symbols. Variables that were significant at *P*<0.05 are shown with solid symbols. NEO, neopterin; TfR, transferrin receptor. Sites are: BGD: Bangladesh – Dhaka; INV: India – Vellore; NEB: Nepal – Bhaktapur; BRF: Brazil – Fortaleza; PEL: Peru – Loreto; SAV: South Africa – Venda; TZH: Tanzania – Haydom.

Supplementary Data

Supplemental Table 1: Variable descriptions and univariate associations with scores on the Wechsler Preschool and Primary Scale of Intelligence for 60-month old children in the MAL-ED project (controlling for site as a random intercept). Univariate models included all available data, but this resulted in different sample size (N children) per model. In the multivariable model the sample size required children with observations for all variables, hence a smaller total number included. Variables with a *p*≤0..2 were retained as candidate variables in the multivariable model.

Variable	β	p.value	include	N
Month of birth	0.020	0.768	FALSE	1200
Infant temperament	0.050	0.202	FALSE	1111
Ever had schooling	2.412	0.001	TRUE	1139
Maternal Factors				
Raven's factor	0.156	0	TRUE	1023
SRQ (60mo, 18 items)	-0.065	0.401	FALSE	1133
SRQ 24mo	-0.165	0.032	TRUE	990
Diet				
Energy per kg body weight 60mo	-0.012	0.212	FALSE	1026
Animal source protein density[1]	0.633	0.068	TRUE	1173
Calcium density[1]	0.929	0.009	TRUE	1173
Cholesterol density[1]	0.294	0.355	FALSE	1173
Carbohydrate density[1]	-1.108	0.167	TRUE	1173
Copper density[1]	0.521	0.127	TRUE	1173
Dairy density[1]	0.703	0.031	TRUE	1173
Mono-unsaturated fat density[1]	0.560	0.232	FALSE	1173
Poly-unsaturated fat density[1]	-0.013	0.971	FALSE	1173
Saturated fat density[1]	1.064	0.009	TRUE	1173
Fat density[1]	0.967	0.058	TRUE	1173
Iron density[1]	0.652	0.102	TRUE	1173
Folate density[1]	0.362	0.345	FALSE	1173
Total energy	0.065	0.787	FALSE	1173
Potassium density[1]	1.101	0.049	TRUE	1173
Meat/fish/poultry iron density[1]	0.024	0.922	FALSE	1173
Meat/fish/poultry protein density[1]	0.226	0.370	FALSE	1173
Magnesium density[1]	0.219	0.754	FALSE	1173
Manganese density[1]	-0.592	0.067	TRUE	1173
Sodium density[1]	-0.374	0.309	FALSE	1173
Phytate density[1]	0.004	0.990	FALSE	1173
Protein density[1]	0.607	0.330	FALSE	1173
Phosphorus density[1]	1.052	0.065	TRUE	1173
Vitamin A density[1]	1.216	0.001	TRUE	1173

Supplementary Data

Vitamin B-12 density[1]	0.569	0.087	TRUE	1173
Vitamin B-6 density[1]	-0.181	0.590	FALSE	1173
Vitamin C density[1]	0.644	0.022	TRUE	1173
Vitamin D density[1]	0.915	0.001	TRUE	1173
Vitamin E density[1]	0.539	0.085	TRUE	1173
Zinc density[1]	1.677	0.007	TRUE	1173
Was the child weaned after 24mo	-0.076	0.893	FALSE	1197
Gut function				
Mean LMZ	-0.234	0.530	FALSE	1118
log(AAT)[2]	-0.459	0.583	FALSE	1150
log(MPO)[2]	-0.082	0.914	FALSE	1150
log(NEO)[2]	-1.073	0.174	TRUE	1150
Growth				
Enrolment WAZ lowest tertile	-1.727	0.004	TRUE	1159
Change in WAZ from enrolment to 2m old (*adaptation to extrauterine environment*)	-0.620	0.307	FALSE	1186
WAZ at 60mo	0.425	0	TRUE	1161
Illness & Treatment				
Proportion of days with ALRI (0-24mo)	0.876	0.836	FALSE	1193
Proportion of days with symptoms of vomiting/fever/ALRI/diarrhea or maternally-reported illness (0-24mo)	-2.059	0.243	FALSE	1193
Proportion of days with diarrhoea (0-24mo)	-1.069	0.855	FALSE	1193
Number of days of hospitalisation in 60th month	-2.964	0.305	FALSE	1076
Proportion of days with maternally-reported illness(0-24mo)	-2.666	0.134	TRUE	1193
Proportion of days with symptoms of vomiting/fever/ALRI/diarrhoea (0-24mo)	0.914	0.726	FALSE	1193
Proportion of days with antibiotics (0-24mo)	-6.142	0.158	TRUE	1193
Micronutrient status				
Ever been anaemic (0-24mo)	-1.706	0.003	TRUE	1072
Ever had high plasma TfR (0-24mo)	-1.933	0.001	TRUE	1072
Ever had low plasma ferritin (0-24mo)	-0.843	0.133	TRUE	1072
Ever had low plasma retinol (0-24mo)	-0.219	0.681	FALSE	1072
Ever had low plasma zinc (0-24mo)	-0.802	0.317	FALSE	1072
Anaemia at 60mo	-0.198	0.814	FALSE	987
Haemoglobin concentration 60mo	-0.080	0.720	FALSE	987
AGP[3]	0.010	0.951	FALSE	1040
Plasma ferritin[3]	0.135	0.351	FALSE	1037
Mean haemoglobin (0-24mo)	0.153	0.462	FALSE	1066
Lead[3]	-0.255	0.444	FALSE	681
Plasma retinol[3]	-0.009	0.980	FALSE	1038

Plasma TfR [3]	-1.485	0.001	TRUE	1036
Plasma zinc[3]	-0.565	0.555	FALSE	878
Number out of low retinol and low ferritin and low zinc and anemia	-0.632	0.012	TRUE	1072
Ever had low retinol or low ferritin or low zinc or anaemia	-1.133	0.217	FALSE	1072
Number of anaemic observations (0-24mo)	-0.896	0.002	TRUE	1072
Number of high plasma TfR observations (0-24mo)	-1.533	0	TRUE	1072
Number of low plasma ferritin observations (0-24mo)	-0.590	0.048	TRUE	1072
Number of low plasma retinol observations (0-24mo)	-0.197	0.558	FALSE	1072
Number of low plasma zinc observations (0-24mo)	-0.249	0.562	FALSE	1072
Ever simultaneous deficiency for ferritin/retinol/anaemia	-0.666	0.312	FALSE	1072
Enteropathogens				
A.lumbricodes nondiarrhoeal[4]	-16.19	0.005	TRUE	1158
Adenovirus nondiarrhoeal[4]	-3.229	0.536	FALSE	1158
aEPEC nondiarrhoeal[4]	0.835	0.815	FALSE	1158
Aeromonas nondiarrhoeal[4]	5.885	0.244	FALSE	1158
Astrovirus nondiarrhoeal [4]	-0.052	0.993	FALSE	1158
Mean bacteria detection rate in non-diarrhoeal stools[4]	-2.193	0.011	TRUE	1158
Campylobacter nondiarrhoeal[4]	-4.094	0.003	TRUE	1158
Cryptosporidium nondiarrhoeal[4]	-0.696	0.886	FALSE	1158
EAEC nondiarrhoeal[4]	-2.164	0.209	FALSE	1158
EIEC nondiarrhoeal [4]	7.007	0.269	FALSE	1158
EPEC nondiarrhoeal [4]	0.382	0.933	FALSE	1158
Giardia nondiarrhoeal[4]	0.316	0.841	FALSE	1158
ltETEC nondiarrhoeal[4]	-0.849	0.821	FALSE	1158
Norovirus (GII) nondiarrhoeal [4]	-1.705	0.685	FALSE	1158
Mean protozoa detection rate in non-diarrhoeal stools[4]	0.352	0.813	FALSE	1158
Rotavirus nondiarrhoeal[4]	-1.555	0.838	FALSE	1158
Mean pathogen detection rate in non-diarrhoeal stools[4]	-1.601	0.011	TRUE	1158
STEC nondiarrhoeal[4]	-4.070	0.591	FALSE	1158
stETEC nondiarrhoeal[4]	-3.876	0.486	FALSE	1158
Mean virus detection rate in non-diarrhoeal stools[4]	-1.956	0.397	FALSE	1158
Socio-economic status & Home environment				
HOME emotional factor 24mo	0.039	0.781	FALSE	1125
HOME safety factor 24mo	1.021	0.001	TRUE	1125
HOME cleanliness factor 24mo	1.072	0	TRUE	1125
Mean number of assets	1.137	0	TRUE	1197
Years of maternal education	0.550	0	TRUE	1195
Agricultural land - No	-1.179	0.087	TRUE	1198
Agricultural land - Yes	1.179	0.087	TRUE	1198
Bovids - No	0.309	0.689	FALSE	1198
Bovids - Yes	-0.309	0.689	FALSE	1198

Supplementary Data

Poultry - No	-0.718	0.308	FALSE	1198
Poultry - Yes	0.718	0.308	FALSE	1198
Kitchen - No	-2.131	0	TRUE	1198
Kitchen - Yes	2.131	0	TRUE	1198
Floor - Cement/concrete	-0.097	0.883	FALSE	1198
Floor - Ceramic tiles or vinyl	3.385	0.003	TRUE	1198
Floor - Earth/sand/clay/mud/dung	-1.405	0.079	TRUE	1198
Floor - Other	0.536	0.910	FALSE	1198
Floor - Wood	-7.388	0.202	FALSE	1198
Roof - Brick	2.207	0.026	TRUE	1198
Roof - Metal	-1.061	0.146	TRUE	1198
Roof - Other	2.093	0.018	TRUE	1198
Roof - Slate	0.676	0.934	FALSE	1198
Roof - Thatch	-1.204	0.104	TRUE	1198
Roof - Tiles	-0.590	0.625	FALSE	1198
Roof - Wood	-5.546	0.176	TRUE	1198
Stove - Closed stove with chimney	-2.883	0.620	FALSE	1198
Stove - Electric heaters	0.609	0.542	FALSE	1198
Stove - Gas stove	2.622	0	TRUE	1198
Stove - Kerosene stove	0.203	0.857	FALSE	1198
Stove - Open fire	-2.860	0	TRUE	1198
Stove - Open fire with chimney	-5.861	0.313	FALSE	1198
Stove - Other	-2.250	0.783	FALSE	1198
Defecation wash - Always	0.851	0.105	TRUE	1198
Defecation wash - Never	-0.978	0.304	FALSE	1198
Defecation wash - Rarely	-0.452	0.602	FALSE	1198
Defecation wash - Sometimes	-0.460	0.422	FALSE	1198
Wash before food - Always	1.139	0.036	TRUE	1198
Wash before food - Never	0.378	0.614	FALSE	1198
Wash before food - Rarely	-0.352	0.676	FALSE	1198
Wash fbefore ood - Sometimes	-1.295	0.022	TRUE	1198
Wash after toilet - Always	1.617	0.003	TRUE	1198
Wash after toilet - Never	-2.043	0.042	TRUE	1198
Wash after toilet - Rarely	-0.250	0.831	FALSE	1198
Wash after toilet - Sometimes	-1.261	0.040	TRUE	1198
Drinking water - Other	4.252	0.004	TRUE	1198
Drinking water - Piped into dwelling	-0.961	0.277	FALSE	1198
Drinking water - Piped to yard/plot	0.835	0.261	FALSE	1198
Drinking water - Protected well	-1.309	0.535	FALSE	1198
Drinking water - Public tap/stand pipe	0.206	0.782	FALSE	1198
Drinking water - Surface water	-1.617	0.139	TRUE	1198
Drinking water - Tube well or borehole	-1.164	0.306	FALSE	1198
Drinking water - Unprotected well	-0.149	0.907	FALSE	1198

Water treatment - No	-1.821	0.010	TRUE	1198
Water treatment - Yes	1.821	0.010	TRUE	1198
Mean monthly household income (USD)	0.007	0	TRUE	1197
Mean Water, sanitation, Assets, Maternal education and Income[5]	15.607	0	TRUE	1197

[1] Diet intake variables were collected from 9 to 24 months and are presented as the mean residual from a linear model using total energy, i.e. the mean density of each intake

[2] Fecal biomarkers (myeloperoxides, neopterin and alpha-1-antitripsin) were detrended for age, stool consistency and recent breast milk and fever. Detailed are given in (3)

[3] Concentrations of blood biomarkers were square-root transformed and averaged across all available observations

[4] Pathogen detection rates are the number of positive assays divided by the number of stools analyzed from 0 to 24 months

[5] The socio-economic construct (WAMI) included access to improved water and sanitation, the number of assets, years of maternal education and monthly household income. It is described in detail in (4)

Supplementary Data

Supplemental Table 2: Mean effect of a one unit change in each variable on the WPPSI T-score from selected univariate models (controlling for site) and the final multivariable model showing relationships between early life experiences and cognitive function at five years in children from the MAL-ED project. Note that the multivariable model also controlled for site, but terms are not shown here.

Model	Univariate			Multivariable			
Variable	β	95% CI		β	95% CI		% Var. explained
Raven's factor	0.16	(0.10,	0.21)	0.09	(0.03,	0.15)	2.80
HOME cleanliness factor 24mo	1.07	(0.60,	1.54)	0.60	(0.05,	1.15)	1.43
Mean income (USD)	0.01	(0,	0.01)	1.99	(-0.69,	4.68)	1.32
Years of maternal education	0.55	(0.42,	0.68)	0.27	(0.08,	0.45)	0.63
Mean number of assets	1.14	(0.86,	1.41)	0.64	(0.24,	1.04)	1.25
Proportion of days with maternally-reported illness (0-24mo)	-2.67	(-6.15,	0.82)	-0.25	(-4.00,	3.50)	0.05
Bacterial detection rate in non-diarrhoeal stools[1]	-2.19	(-3.88,	0.50)	-0.33	(-2.33,	1.67)	0.26
log(NEO)[2]	-1.07	(-2.62,	0.47)	-1.16	(-2.94,	0.63)	0.06
Animal source protein density[3]	0.63	(-0.05,	1.31)	0.28	(-0.89,	1.46)	0.22
Vitamin A density[3]	1.22	(0.53,	1.90)	0.34	(-0.74,	1.41)	0.14
Vitamin B-12 density[3]	0.57	(-0.08,	1.22)	-0.32	(-1.47,	0.84)	0.02
Vitamin D density[3]	0.92	(0.39,	1.44)	0.42	(-0.23,	1.08)	0.31
Zinc density[3]	1.68	(0.45,	2.90)	0.51	(-1.31,	2.34)	0.02
Plasma TfR[4]	-1.48	(-2.34,	-0.63)	-1.81	(-2.75,	-0.86)	0.95

[1] Pathogen detection rates are the number of positive assays divided by the number of stools analyzed from 0 to 24 months
[2] Fecal biomarkers (myeloperoxides, neopterin and alpha-1-antitripsin) were detrended for age, stool consistency and recent breast milk and fever. Detailed are given in (3)
[3]Diet intake variables were collected from 9 to 24 months and are presented as the mean residual from a linear model using total energy, i.e. the mean density of each intake
[4] Concentrations of blood biomarkers were square-root transformed and mean-averaged across all available observations

Supplementary Data

Supplemental Table 3: Mean effect of a one unit change in each variable on the WPPSI T-score comparing the final multivariable model and models using the total detection of enteropathogens (rather than bacterial detections) and the number of bacterial detections using the TAC qPCR in children from the MAL-ED project.

Model	Multivariable			Total pathogens			TAC bacteria		
**Variable**	**β**	**95% CI**		**β**	**95% CI**		**β**	**95% CI**	
Mean number of assets	0.64	(0.24	1.04)	0.65	(0.25	1.05)	0.65	(0.25	1.06)
Bacteria detection rate in non-diarrhoeal stools[1]	-0.33	(-2.33	1.67)	NA	NA	NA	NA	NA	NA
Pathogen detection rate in non-diarrhoeal stools[1]	NA	NA	NA	0.19	(-1.26	1.64)	NA	NA	NA
TAC qPCR bacterial detection in non-diarrhoeal stools[1b]	NA	NA	NA	NA	NA	NA	-0.01	(-1.55	1.52)
Years of maternal education	0.27	(0.08	0.45)	0.27	(0.09	0.45)	0.25	(0.07	0.43)
Raven's factor	0.09	(0.03	0.15)	0.09	(0.03	0.15)	0.10	(0.04	0.16)
HOME cleanliness factor 24mo	0.60	(0.05	1.15)	0.60	(0.05	1.15)	0.60	(0.05	1.15)
Proportion of days with maternally-reported illness (0-24mo)	-0.25	(-4.00	3.50)	-0.23	(-3.98	3.53)	-0.22	(-4.02	3.57)
Mean income (USD)	1.99	(-0.69	4.68)	2.02	(-0.67	4.70)	2.16	(-0.55	4.86)
Plasma TfR[2]	-1.81	(-2.75	-0.86)	-1.82	(-2.77	-0.88)	-1.74	(-2.68	-0.79)
log(NEO)[3]	-1.16	(-2.94	0.63)	-1.16	(-2.94	0.63)	-0.89	(-2.68	0.91)
Animal source protein density[4]	0.28	(-0.89	1.46)	0.29	(-0.88	1.47)	0.19	(-1.01	1.38)
Vitamin A density[4]	0.34	(-0.74	1.41)	0.34	(-0.73	1.42)	0.32	(-0.77	1.40)
Vitamin B-12 density[4]	-0.32	(-1.47	0.84)	-0.33	(-1.48	0.83)	-0.32	(-1.49	0.85)
Vitamin D density[4]	0.42	(-0.23	1.08)	0.44	(-0.22	1.10)	0.46	(-0.20	1.12)
Zinc density[4]	0.51	(-1.31	2.34)	0.50	(-1.32	2.32)	0.94	(-0.89	2.76)

[1] Pathogen detection rates are the number of positive assays divided by the number of stools analyzed from 0 to 24 months
[2] Concentrations of blood biomarkers were square-root transformed and mean-averaged across all available observations
[3] Fecal biomarkers (myeloperoxides, neopterin and alpha-1-antitripsin) were detrended for age, stool consistency and recent breast milk and fever. Detailed are given in (3)
[4] Diet intake variables were collected from 9 to 24 months and are presented as the mean residual from a linear model using total energy, i.e. the mean density of each intake
[b] TAC re-analysis of samples used quantitative PCR as detailed in (5)

Supplemental Table 4: Mean effect of a one unit change in each variable on the WPPSI T-score in three sub-analyses run separately due to diminished sample sizes: (i) including whether the mother had depressive symptoms (SRQ), (ii) whether the child had ever had schooling prior to the WPPSI assessment and (iii) including the mean hemoglobin from blood drawn between 0 and 24 months in children from the MAL-ED project.

**Model**	**Maternal depression**			**Any schooling**			**Hemoglobin**		
**Variable**	**β**	**95% CI**		**β**	**95% CI**		**β**	**95% CI**	
Mean number of assets	0.69	(0.29	1.08)	0.64	(0.24	1.05)	0.63	(0.23	1.03)
Bacteria detection rate in non-diarrhoeal stools[1]	-0.54	(-2.63	1.56)	-0.45	(-2.48	1.58)	-0.30	(-2.30	1.71)
Years of maternal education	0.29	(0.11	0.47)	0.28	(0.09	0.46)	0.27	(0.09	0.45)
Ever had schooling	NA	NA	NA	1.51	(-0.14	3.16)	NA	NA	NA
Raven's factor	0.06	(0	0.12)	0.09	(0.02	0.15)	0.09	(0.03	0.15)
HOME cleanliness factor 24mo	0.53	(-0.02	1.07)	0.5	(-0.07	1.06)	0.60	(0.05	1.15)
Proportion of days with maternally-reported illness (0-24mo)	-0.22	(-3.91	3.48)	-0.13	(-3.89	3.63)	-0.27	(-4.03	3.49)
Mean income (USD)	1.85	(-0.79	4.49)	1.84	(-0.87	4.55)	2.05	(-0.65	4.74)
Hemoglobin[2]	NA	NA	NA	NA	NA	NA	-1.19	(-4.43	2.04)
Plasma TfR[2]	-1.85	(-2.80	-0.90)	-1.87	(-2.82	-0.92)	-1.94	(-2.97	-0.91)
log(NEO)[3]	-1.06	(-2.87	0.74)	-1.50	(-3.33	0.32)	-1.21	(-3.01	0.59)
Animal source protein density[4]	0.23	(-0.97	1.44)	0.28	(-0.90	1.46)	0.26	(-0.92	1.45)
Vitamin A density[4]	0.44	(-0.64	1.51)	0.29	(-0.80	1.38)	0.31	(-0.77	1.39)
Vitamin B-12 density[4]	-0.21	(-1.37	0.95)	-0.31	(-1.47	0.85)	-0.29	(-1.45	0.88)
Vitamin D density[4]	0.25	(-0.41	0.91)	0.39	(-0.28	1.06)	0.44	(-0.21	1.10)
Zinc density[4]	-0.29	(-2.28	1.71)	0.68	(-1.16	2.51)	0.49	(-1.35	2.33)
SRQ 24mo	-0.10	(-0.27	0.07)	NA	NA	NA	NA	NA	NA

[1] Pathogen detection rates are the number of positive assays divided by the number of stools analyzed from 0 to 24 months
[2] Concentrations of blood biomarkers (not hemoglobin) were square-root transformed and mean-averaged across all available observations
[3] Fecal biomarkers (myeloperoxides, neopterin and alpha-1-antitripsin) were detrended for age, stool consistency and recent breast milk and fever. Detailed are given in (3)
[4] Diet intake variables were collected from 9 to 24 months and are presented as the mean residual from a linear model using total energy, i.e. the mean density of each intake

Supplementary Data

Supplemental Table 5: Comparison of the final multivariable model and models imputing missing observations (using MICE) and for over-fitting using LASSO regression in children from the MAL-ED project.

Model	Multivariable (n=813)			MICE (n=1198)			LASSO (n=813)		
**Variable**	**β**	**95% CI**		**β**	**95% CI**		**β**	**95% CI**	
Mean number of assets	0.64	(0.24	1..04)	0.57	(0.23	0.91)	0.64	(0.27	0.98)
Bacteria detection rate in non-diarrheal stools[1]	-0.33	(-2.33	1.67)	-1.26	(-3.01	0.48)	0	(-1.91	1.87)
Years of maternal education	0.27	(0.08	0.45)	0.23	(0.07	0.39)	0.27	(0.10	0.47)
Raven's factor	0.09	(0.03	0.15)	0.08	(0.02	0.14)	0.09	(0.03	0.14)
HOME cleanliness factor 24mo	0.60	(0.05	1.15)	0.50	(0.05	0.96)	0.60	(0.03	1.16)
Proportion of days with maternally-reported illness (0-24mo)	-0.25	(-4.00	3.50)	-0.93	(-4.31	2.46)	0	(-3.52	3.49)
Mean income (USD)	1.99	(-0.69	4.68)	1.58	(-0.57	3.72)	2.05	(-0.56	4.09)
Plasma TfR[2]	-1.81	(-2.75	-0.86)	-1.20	(-2.06	-0.35)	-1.82	(-2.75	-0.85)
log(NEO)[3]	-1.16	(-2.94	0.63)	-1.36	(-2.88	0.16)	-1.14	(-3.05	0.49)
Animal source protein density[4]	0.28	(-0.89	1.46)	-0.03	(-1.02	0.96)	0	(-0.94	1.07)
Vitamin A density[4]	0.34	(-0.74	1.41)	0.70	(-0.18	1.59)	0.30	(-0.78	1.36)
Vitamin B-12 density[4]	-0.32	(-1.47	0.84)	-0.26	(-1.23	0.71)	0	(-1.04	1.03)
Vitamin D density[4]	0.42	(-0.23	1.08)	0.20	(-0.34	0.74)	0.40	(-0.27	0.81)
Zinc density[4]	0.51	(-1.31	2.34)	0.74	(-0.67	2.16)	0.54	(-1.06	2.25)

To impute data, 20 datasets constructed using multiple imputation chain equations and predictive mean matching (selecting from 10 closest observations) based on the other variables selected in the final model. The LASSO regularization parameter was identified using one standard deviation from the optimum (minimum mean error) derived from 10-fold cross-validation with boot-strap derived confidence intervals.

[1] Pathogen detection rates are the number of positive assays divided by the number of stools analyzed from 0 to 24 months

[2] Concentrations of blood biomarkers were square-root transformed and mean-averaged across all available observations

[3] Fecal biomarkers (myeloperoxides, neopterin and alpha-1-antitripsin) were detrended for age, stool consistency and recent breast milk and fever. Detailed are given in (3)

[4] Diet intake variables were collected from 9 to 24 months and are presented as the mean residual from a linear model using total energy, i.e. the mean density of each intake

Supplementary Data

Supplemental References:

1. MAL-ED Network Investigators. Early childhood cognitive development is affected by interactions among illness, diet, enteropathogens and the home environment: findings from the MAL-ED birth cohort study. BMJ Global Health. 2018;3:e000752.
2. Greenland S, Pearl J, Robins JM. Causal diagrams for epidemiologic research. Epidemiology 1999;10:37–48.
3. McCormick BJJ, Lee GO, Seidman JC, *et al.* Dynamics and trends in fecal biomarkers of gut function in children from 1–24 months in the MAL-ED study. *Am J Trop Med Hyg* 2017; 96(2):465-472.
4. Psaki SR, Seidman JC, Miller M, Gottlieb M, Bhutta ZA, Ahmed T, Ahmed AS, Bessong P, John SM, Kang G, et al. Measuring socioeconomic status in multicountry studies: results from the eight-country MAL-ED study. Popul Health Metr. 2014;12:8.
5. Liu J, Kabir F, Manneh J, Lertsethtakarn P, Begum S, Gratz J, Becker SM, Operario DJ, Taniuchi M, Janaki L, et al. Development and assessment of molecular diagnostic tests for 15 enteropathogens causing childhood diarrhoea: a multicentre study. Lancet Infect Dis. 2014;14:716–24.
